# Managing blunt cardiac injury

**DOI:** 10.1186/s13019-023-02146-z

**Published:** 2023-02-10

**Authors:** Lawrence Nair, Brendan Winkle, Eshan Senanayake

**Affiliations:** 1 Department of Cardiothoracic Surgery, The Prince Charles Hospital, Rode Road, Chermside, Brisbane, QLD 4032 Australia; 2 Department of Surgery, Royal Darwin Hospital, Darwin, NT Australia; 3 Department of Cardiothoracic Surgery, Queen Elizabeth Hospital, Birmingham, United Kingdom

**Keywords:** Blunt cardiac injury, Contusion, Rupture, Cardiorrhaphy

## Abstract

Blunt cardiac injury (BCI) encompasses a spectrum of pathologies ranging from clinically silent, transient arrhythmias to deadly cardiac wall rupture. Of diagnosed BCIs, cardiac contusion is most common. Suggestive symptoms may be unrelated to BCI, while some injuries may be clinically asymptomatic. Cardiac rupture is the most devastating complication of BCI. Most patients who sustain rupture of a heart chamber do not reach the emergency department alive. The incidence of BCI following blunt thoracic trauma remains variable and no gold standard exists to either diagnose cardiac injury or provide management. Diagnostic tests should be limited to identifying those patients who are at risk of developing cardiac complications as a result of cardiac in jury. Therapeutic interventions should be directed to treat the complications of cardiac injury. Prompt, appropriate and well-orchestrated surgical treatment is invaluable in the management of the unstable patients.

## Background

Blunt cardiac injury (BCI) encompasses a spectrum of pathologies ranging from clinically silent, transient arrhythmias to deadly cardiac wall rupture. Therefore, the real incidence of BCI is difficult to estimate and can vary widely. Of diagnosed BCIs, cardiac contusion is most common. The absence of clear diagnostic criteria and reliable diagnostic tests makes reporting difficult. Suggestive symptoms may be unrelated to BCI, while some injuries may be clinically asymptomatic. Cardiac rupture is the most devastating complication of BCI. Most patients who sustain rupture of a heart chamber do not reach the emergency department alive.

BCI occurs most often from motor vehicle collisions (MVCs). Rapid deceleration is the mechanism responsible for most BCIs. A direct blow to the precordium also accounts for a sizable number of cases [1, 2]. Any patient involved in an MVC with sudden deceleration, or who sustains significant chest trauma or severe multiple trauma is at risk.

Several forces may be involved in BCI, including compression of the heart between the spine and sternum, abrupt pressure fluctuations in the chest and abdomen, shearing from rapid deceleration, and blast injury [2]. In addition, fragments from rib fractures can directly traumatize the heart without a penetrating injury. The right heart is the most commonly injured, likely due to its position closest to the anterior chest wall. High pressure ventricular injuries appear to be as common as low pressure atrial injuries. Other pathological findings include valvular tears or rupture, septal tears and coronary artery thrombosis or laceration, however these are less common [2].

### Types of injury

#### Cardiac contusion

Cardiac contusions are the most common injuries to the heart resulting from blunt trauma. Mild cardiac contusions often recover without lasting consequences while severe injuries more often result in lasting consequences and mortality [3].

Histologically, it is characterized by a contused myocardium with haemorrhagic infiltrate, localised necrosis, and oedema.

Signs and symptoms of cardiac contusions include chest pain, shortness of breath, and the development of arrhythmias. Cardiac contusions are often difficult to accurately diagnose due to the lack of standardised approaches to their evaluation.

Since cardiac contusion defines a wide range of injuries, patients may have a variety of presentations. Patients presenting with cardiac contusions range from being completely asymptomatic to experiencing mild chest soreness, presenting with electrocardiographic abnormalities, contractile abnormalities, and having signs of heart failure.

Right bundle branch block (RBBB) is a common result of cardiac contusions, whilst left bundle branch block (LBBB) has rarely been reported [4]. The right ventricle is nearest to the sternum, subjecting it to the high risk of cardiac contusions [4].

As there is often variability in severity and presentation of cardiac contusions, various diagnostic modalities have been utilised to aid the evaluation and treatment process. Cardiac contusions are characterised by injury to the myocardium. Cardiac enzymes such as creatinine kinase and troponin, enzymes that are released after an injury to the heart, and have traditionally been used in evaluating patients for MI. They have also been utilised in the evaluation of cardiac trauma as both can be increased due to injury of the heart [3].

Imaging can be utilised to identify structural damage to the heart after BCI. Echocardiography provides functional and structural assessments of the heart and is important for ruling out other injuries to the heart including valvular dysfunction, septal or free wall rupture, cardiac tamponade, and muscle function. Cardiac magnetic resonance imaging (MRI) is an alternative option for the diagnosis of cardiac abnormalities with the ability to provide information regarding the extent of myocardial contusion and regional infarcts.

#### Myocardial rupture

Nonspecific signs and concomitant injuries make the clinical diagnosis of blunt myocardial injury difficult. Signs such as hypotension associated with distended neck veins and muffled heart sounds suggest pericardial tamponade, which may occur with BCI. However, such signs may not be present; the patient with haemorrhage and hypotension may not have distended neck veins; in this case an immediate bedside ultrasound may reveal the diagnosis.

Most patients with severe BCI, such as uncontained myocardial rupture, do not reach the emergency department alive [5]. Of those who do, hypotension may reduce pressure on the injured myocardium, which in turn may then worsen as fluid resuscitation restores blood pressure and subsequent increase in myocardial pressure increases. In a minority of patients, rapid diagnosis by echocardiography or CT scan and operative intervention can be lifesaving [6].

Atrial rupture occurs far less often than ventricular rupture, likely due to the location of the atria and their compliance. Atrial injuries tend to be delayed in presentation and often present less acutely [5]. Right atrial rupture is seen in approximately 10% of wall ruptures from blunt trauma, with left atrial rupture less common [7].

#### Septal and valvular injury

Septal injury appears to be rare, and its presentation varies. Septal injury may involve insignificant tears or frank rupture, and may occur in isolation or with valvular injury [2]. Findings may include acute valvular insufficiency with widened pulse pressure and signs of acute heart failure.

Isolated valvular injury is likewise rare [8]. The aortic valve is most often injured, followed by the mitral and tricuspid valves [8]. The lesion may consist of a tear of the leaflet or a partial or full thickness tear of the papillary muscle or chordae tendineae. Presentation may vary, in part depending upon the lesion, but falls somewhere in the spectrum of acute valvular insufficiency with right-or left-sided heart failure and a new cardiac murmur.

Ismailov and colleagues noted a significant increase in tricuspid and aortic valve insufficiency, incompetence, and regurgitation among patients with a history of BCI. Milder cases may go undiagnosed initially and present late with heart failure from long-standing valvular dysfunction [9]. While less common, mitral valve insufficiency has also been reported [10].

#### Concomitant injury and sternal fracture

BCI often presents with concomitant injuries. These can include injuries to the head, thorax, abdomen, and spine. In one autopsy series, sternal fractures were found in 76% of cases involving cardiac injury but only 18% of deaths without BCI [11]. Nevertheless, a sternal fracture does not necessarily imply the presence of BCI, but a high degree of suspicion for blunt cardiac injury should be considered and excluded based on the mechanism of injury.

#### Myocardial infarction

Myocardial infarction is a rare complication of BCI reported in victims of motor vehicle collisions and minor trauma. Causes include coronary artery dissection, laceration, and thrombosis [12, 13]. The left anterior descending artery appears to be involved most often [14].

## Discussion

The exact incidence of BCI is unknown. The cause of cardiac dysfunction following BCI can be difficult to determine in the injured patients who possess many reasons for hypotension and haemodynamic instability. Rupture of a cardiac chamber after nonpenetrating thoracic trauma has a high mortality rate, with most patients dying at the scene of the accident. Such injuries, however, are now being recognized with increasing frequency, and the potential for survival exists in patients who are rapidly transported to trauma centres [15].

The biomechanics of cardiac chamber rupture after blunt thoracic trauma are thought to be secondary to rapid deceleration or direct precordial impact [16]. Survival after single-chamber cardiac rupture is more likely with atrial than ventricular ruptures and with injuries involving the right heart chambers [17, 18]. Rupture of the atria, frequently noted at the junction of the atrium with the vena cava or pulmonary veins, is thought to be associated with rapid deceleration [18]. It has been suggested that these structures have different rates of deceleration related to their degree of fixation in the mediastinum, leading to shearing forces generated during rapid and sudden deceleration [19]. It has been previously proposed that right atrial ruptures are the most common secondary to the weakness of the right atrial appendage [20]; however, Parmely and colleagues demonstrated an equal frequency of rupture for all four cardiac chambers [8]. By contrast, ventricular rupture appears more likely to occur after a direct injury to the precordium, resulting in compression of the heart between the sternum and the vertebral column. Rupture is more likely to occur at end-diastole, when the ventricle is maximally distended and the force of ventricular compression greater [21].

It has been suggested that survival may be improved in the presence of a pericardial tear, as immediate cardiac tamponade may be avoided [22]. However, decompression into the pleural space would result in massive haemothorax, and subsequent mortality is also likely to be high.

Patient presentation and clinical findings will ultimately determine management. If a patient is indeed able to survive the initial trauma and arrive at a medical centre equally capable of providing treatment, prompt, coordinated and appropriate surgical intervention proves to be the best predictor of patient survival.

Control of the ruptured myocardium is usually achieved by direct suture after digital pressure, application of a vascular clamp, or the use of prosthetic material anchored with surgical glue. However, the use of cardiopulmonary bypass has been reported should such measures be unsuccessful and available.

A well-coordinated plan for stemming massive blood loss in conjunction with known surgical techniques for repair of ventricular disruption provides the initial foundation for the surgical treatment. It is imperative that patients with clinical or echocardiographic evidence of severe cardiac injury, i.e., ruptured valve, septum, or ventricular wall causing cardiac tamponade, receive emergent surgical consultation. If tamponade is suspected either clinically or by ultrasound, pericardiocentesis can be performed. Tamponade that results from an atrial tear may be amenable to pericardiocentesis with periodic drainage using a pigtail catheter until definitive surgical repair can be performed. Among unstable patients who may not survive transfer to an operating room, emergency department thoracotomy, rather than pericardiocentesis, may be the best treatment for cardiac tamponade. Nevertheless, in the setting of blunt trauma, ED thoracotomy rarely results in successful resuscitation.

Even following prompt diagnosis, subsequent surgical management remains quite challenging for several reasons. First, patients generally have marked hemodynamic instability and are thus considered to be an extremely high-risk surgical candidates. Induction agents with cardio-depressive effects and positive pressure ventilation following intubation may further compromise cardiac function in patients with these injuries. If possible, it may be beneficial to delay intubation until the patient is in the operating room. The results from available case series in the literature regarding patients who undergo surgery suggest that the degree of preoperative haemodynamic instability may be the most important predictor of outcome, with survival rates ranging from 39 to 100% [23].

Regarding surgery, the location of the rupture site can be very elusive and paradoxically aggravated by the use of cardiopulmonary bypass [24]. In an effort to ease identification of the culprit region of myocardium, surgical approaches are now frequently being performed off-pump [24]. Following identification of the lesion, well-performed cardiorrhaphy and appropriate selection and use of surgical materials will aid in the acute surgical management. Noncutting sutures used in a buttress technique plus the use of artificial patches and glues have been well documented in providing adequate closure options [15, 25]. The major technical issue with the surgical repair of a rupture involves the weakened state of the surrounding myocardium, where the weakened tissue serves as a very poor scaffold site for suturing. Contemporary techniques have overcome this issue through the use of a Dacron or Teflon patches, which are large enough to cover the area of rupture and surrounding weakened tissue and incorporate the neighbouring viable myocardium [24–26], as also noted in ruptures post infarct. The patch, which can be applied without sutures using cyanoacrylate glue, results in a stable seal that stops the myocardial leak while preserving the original left ventricular cavity size. The off-pump sutureless patch technique appears to be the favoured surgical approach for the management of left ventricular free wall rupture.

## Conclusion

The incidence of BCI following blunt thoracic trauma remains variable and no gold standard exists to either diagnose cardiac injury or provide management.

Diagnostic tests should be limited to identifying those patients who are at risk of developing cardiac complications as a result of cardiac in jury. Therapeutic interventions should be directed to treat the complications of cardiac injury. Prompt, appropriate and well-orchestrated surgical treatment is invaluable in the management of the unstable patient.

## Data Availability

Not applicable.
